# HIF-2α Expression Regulates Sprout Formation into 3D Fibrin Matrices in Prolonged Hypoxia in Human Microvascular Endothelial Cells

**DOI:** 10.1371/journal.pone.0160700

**Published:** 2016-08-04

**Authors:** Tessa D. Nauta, Monique C. A. Duyndam, Ester M. Weijers, Victor M. W. van Hinsbergh, Pieter Koolwijk

**Affiliations:** 1 Department of Physiology, Institute for Cardiovascular Research, VU University Medical Center, De Boelelaan 1118 1081 HV, Amsterdam, The Netherlands; 2 A-Skin Nederland BV, De Boelelaan 1117, Amsterdam 1007 MB, The Netherlands; University of Illinois at Chicago, UNITED STATES

## Abstract

**Background:**

During short-term hypoxia, Hypoxia Inducible Factors (particular their subunits HIF-1α and HIF-2α) regulate the expression of many genes including the potent angiogenesis stimulator VEGF. However, in some pathological conditions chronic hypoxia occurs and is accompanied by reduced angiogenesis.

**Objectives:**

We investigated the effect of prolonged hypoxia on the proliferation and sprouting ability of human microvascular endothelial cells and the involvement of the HIFs and Dll4/Notch signaling.

**Methods and Results:**

Human microvascular endothelial cells (hMVECs), cultured at 20% oxygen for 14 days and seeded on top of 3D fibrin matrices, formed sprouts when stimulated with VEGF-A/TNFα. In contrast, hMVECs precultured at 1% oxygen for 14 days were viable and proliferative, but did not form sprouts into fibrin upon VEGF-A/TNFα stimulation at 1% oxygen. Silencing of HIF-2α with si-RNA partially restored the inhibition of endothelial sprouting, whereas HIF-1α or HIF-3α by si-RNA had no effect. No involvement of Dll4/Notch pathway in the inhibitory effect on endothelial sprouting by prolonged hypoxia was found. In addition, hypoxia decreased the production of urokinase-type plasminogen activator (uPA), needed for migration and invasion, without a significant effect on its inhibitor PAI-1. This was independent of HIF-2α, as si-HIF-2α did not counteract uPA reduction.

**Conclusion:**

Prolonged culturing of hMVECs at 1% oxygen inhibited endothelial sprouting into fibrin. Two independent mechanisms contribute. Silencing of HIF-2α with si-RNA partially restored the inhibition of endothelial sprouting pointing to a HIF-2α-dependent mechanism. In addition, reduction of uPA contributed to reduced endothelial tube formation in a fibrin matrix during prolonged hypoxia.

## Introduction

Angiogenesis is important for growth, development and proper wound healing, but is also associated with several pathological conditions, such as tissue ischemia, solid tumors, rheumatoid arthritis and adult macular degeneration of the eye [1]. Usually, these disorders are accompanied by loss of adequate blood supply or enhanced metabolic demand, leading to reduced oxygen tension (hypoxia) in the tissue. Not surprisingly, hypoxia is considered to be one of the most potent initiators of angiogenesis *in vitro* and *in vivo* [2–4]. Despite the impact of hypoxia on continuing angiogenesis in tumors and expanding tissues, prolonged hypoxia in poorly perfused or healing tissues is often accompanied by a resistance to neovascularization [3,5–8]. Limited expression of Hypoxia Inducible Factor-1 (HIF-1) and HIF-induced angiogenic factors has been observed in chronically hypoxic human leg muscle tissue at the time of limb amputation [9]. Resistance to vascularization may also become problematic when a tissue-engineered graft is implanted in the body. The graft soon encounters a severe and cell damaging hypoxia, which can only be counteracted by rapid restoration of perfusion, i.e. induction of neovascularization and connection to the circulating blood [10–13]. While angiogenesis may be induced at the border between graft and perfused host tissue, it does often not penetrate adequately into the grafted tissue. Therefore, there is a need of overcoming endogenous inhibitory factors that prevent induction of angiogenesis in severely hypoxic tissues.

Despite the progress in knowledge on inhibiting angiogenesis [14,15], little is known about the stimulation of angiogenesis during long-term hypoxia. Immediately upon hypoxia exposure HIF-1α is stabilized in cells and subsequently transferred to the nucleus, where it forms the transcription factor HIF-1 and induces the expression of many genes including VEGF-A. Exposure of endothelial cells to high concentrations of VEGF-A and other angiogenic growth factors induces tortuous and leaky newly formed vascular structures that are not adequately perfused [16,17]. However, besides the initiation of the angiogenesis process by the HIF-1α-mediated VEGF/VEGF receptor pathway [18,19], the response of endothelial cells to hypoxia is more complex and involves the activation of both HIF-1α and HIF-2α. The balance between HIF-1α and HIF-2α has been proposed as a regulator of (excessive) sprouting and (extensive) elongation of new endothelial structures [20]. Endothelial-specific deletion of HIF-2α pointed to a role of HIF-2α in the regulation of angiogenesis in mouse lungs. Moreover, HIF-2α was indicated as an important regulator of Dll4/Notch signaling pathway by hypoxia, thus modulating endothelial sprouting in mouse lungs [20,21]. Recently Gong *et al* [22] reported that HIF-2α was involved in hypoxia-induced improvement of the endothelial barrier function of lung endothelial cells, a process involving VE-cadherin and the tyrosine phosphatase VT-PTP. These data were obtained in short-term hypoxia, i.e. evaluation occurred within 1–2 days. Alternatively, Ginouvès *et al* [23], who evaluated prolonged hypoxia, suggested that downregulation of HIF-1α and HIF-2α occurs after prolonged (7 days) hypoxia by upregulation and over-activation of prolyl dehydrogenases (PHDs), in particular to prevent HIF-induced apoptosis and cell death. This also may affect the induction of angiogenesis. These findings indicate a need for better understanding the effect of prolonged hypoxia on sprouting angiogenesis and the role of HIF-2α therein.

Previously we have described a model to study the formation of endothelial tubes by human microvascular endothelial cells (hMVECs) in 3D fibrin matrices [24]. Endothelial tube formation required angiogenic growth factors, enhanced by TNFα and was dependent on pericellular proteolysis generated by the urokinase/plasmin system [24–26] or MMP14 [27–31]. Similar as other investigators [2,4,32], we observed previously a stimulation of tube formation in short-term hypoxia [33]. Subsequently we created a method to study endothelial behavior in prolonged hypoxia by using a hypoxic workstation that allowed handling and culture medium refreshment of the cells in a continuous low oxygen atmosphere. Under these conditions a severely hampered endothelial tube formation by hMVECs was observed in prolonged hypoxia. Here we report on these observations and demonstrate that inhibition of HIF-2α expression, but not HIF-1α, can partly overcome the tube forming inhibition caused by prolonged hypoxia.

## Material and Methods

### Cell culture

The study was executed in accordance with the Declaration of Helsinki and was approved by the University Human Subjects Committee of the VU University Medical Center. Written informed consent was obtained from all donors in accordance with the institutional guidelines. Human microvascular endothelial cells (hMVECs) were isolated from foreskin, kindly provided by the Department of Dermatology (VUmc, Amsterdam), cultured and characterized (CD31, vWF, Ulex europaeus lectin-1 binding, VE-cadherin) as previously described [34,35]. hMVECs were cultured on 1% gelatin-coated culture plates in culture medium consisting of Medium 199 supplemented with 100 U/ml penicillin and 100 mg/ml streptomycin (p/s), 2 mM L-glutamine (all Lonza, Verviers, Belgium), 5 U/ml heparin (Leo Pharmaceutical Products, Weesp, The Netherlands), endothelial cell growth factor (ECGF, crude extract from bovine brain), 10% heat-inactivated human serum (HSi, PAA Laboratories, Pasching, Austria) and 10% heat-inactivated newborn calf serum (NBCSi, Lonza). Medium was changed every 48 hours. Confluent cells were washed with 0.5 mM EDTA (Merck Millipore) in HBSS, trypsinized (0.05% trypsin in EDTA/HBSS, Lonza) and seeded in a 1:3 density. Cells were cultured at 37°C in a water-saturated atmosphere of 95% air and 5% CO_2_. hMVECs were used until passage 10. Human lung microvascular endothelial cells (lung hMVECs) were cultured and characterized as previously described [35].

### Hypoxic cell culture

Hypoxic cell culture conditions were maintained inside a custom designed hypoxic workstation (T.C.P.S., Rotselaar, Belgium), with CO_2_ and O_2_ controlled (via injection of N_2_), humidified incubators (Sanyo, Ettenleur, The Netherlands), placed inside a T4 glovebox (Jacomex, Dagneux, France) equipped with an O2X1 oxygen transmitter (GE Panametrics, Billerica, USA). The oxygen concentration inside the incubators were continuously monitored with an internal zirconia sensor and periodically checked with O_2_ test tubes (Drager Safety, Zoetermeer, The Netherlands). To prevent re-oxygenation during hypoxic culturing, all media and buffers were transferred into the hypoxic workstation through a lock and pre-incubated for 4h before use. For the long-term hypoxic culture of hMVECs, isolates were cultured for 2 passages (~14 days) inside the hypoxic workstation.

### PreSens Oxo Dish

Medium containing 20% oxygen was added to a 24-wells Oxo Dish^®^ (PreSens, Regensburg, Germany), which contains a sensor spot at the bottom of each well, in the hypoxic workstation. With 5 minute intervals, the decrease in oxygen concentration was measured with a SDR SensorDish^®^ Reader (PreSens) connected to a computer. The increase in oxygen concentration after addition of 1% oxygen-containing medium to a 24-wells Oxo Dish^®^ in atmospheric air followed a similar procedure.

### *In vitro* tube formation assay

3D human tube formation was evaluated as previously described [24]. 2 mg/mL fibrinogen (Stago bnl Leiden, The Netherlands) was dissolved in M199 medium + p/s. Thrombin (0.05 U/mL) was added to the fibrinogen solution and 100 μL was immediately added to the wells of a 96-well plate. For polymerization, plates were incubated for one hour at room temperature followed by one hour at 37°C. Thrombin was inactivated by addition of serum-supplemented culture (SSC) medium consisting of Medium 199 with p/s supplemented with 10% HSi, 10% NBCSi and 2 mM L-glutamine. hMVECs, precultured for 14 days at 20% or 1% oxygen, were seeded in a confluent density on top of the fibrin matrices. After 24 hours, and subsequently at 48h intervals, the hMVECs were stimulated with SSC medium with 10 ng/ml tumor necrosis factor-α (TNFα, Sigma, St Louis, USA) and 25 ng/ml Vascular Endothelial Growth Factor (VEGF, Invitrogen, Carlsbad, USA) or 10 ng/ml TNFα and 10 ng/ml basic fibroblast growth factor (bFGF, Preprotech, London, UK). The experiments were terminated by fixation with 2% paraformaldehyde/HBSS for two hours at room temperature. The formation of tube-like structures into the fibrin matrices was analyzed by phase contrast microscopy and Optimas image analysis software (Media Cybernetics).

When indicated, human recombinant uPA (hr-uPA; equivalent to 174,000 IU/mg; a gift from Dr Willem Nieuwenhuizen, Gaubius Institute Leiden) was added in different concentrations (from 2.5 to 20 ng/mL) to the SCC medium with VEGF and TNFα.

### ELISA

For enzyme-linked immunosorbent assay (ELISA) determination of soluble uPA and PAI-1 antigens, supernatants were collected from the 3D tube formation assay 72 hours after stimulation and assayed as previously described [24,36]. The monoclonal uPAR-blocking antibody H-2 came from Boehringer Mannheim, Penzberg, Germany [37] and was used to determine the overall internalization of the uPA:PAI-1 complex by blocking the uPA binding to uPAR, as previously described [26].

### Transfection method

30x10^4^ hMVECs (on 10 cm^2^) were transfected with 25 nM of indicated si-RNA (si-HIF-1α and si-HIF-2α were custom designed and obtained from Qiagen (Venlo, The Netherlands) and si-HIF-3α, si-uPA, si-uPAR, si-PAI-1 were ordered as FlexiTube from Qiagen) using DharmaFECT (GE Dharmacon Lafayette, CO). In short; hMVECs were transfected with 2 mL 10% HSi/M199 containing 2.5 μL DharmaFECT transfection reagent Type 1 and si-RNA. 18 hours after transfection, cells were refreshed with culture medium to start experiment.

### Proliferation Assay

hMVECs were seeded in a density of 10x10^3^ cells/cm^2^ on 1% gelatin-coated plates in 20% and 1% oxygen conditions (in triplicate wells for each condition). Every day phase-contrast pictures were taken with a Qimaging camera on a Zeiss microscope connected to a computer with Optimas image analysis software (Media Cybernetics). ImageJ was used to count the number of cells. Inside the hypoxic workstation, the camera was attached to the phase-contrast microscope, and a similar procedure was followed to determine the hMVEC proliferation. In addition, cell division was quantified after incorporation with 5-ethynyl-2’-deoxyuracil (EdU) (Invitrogen) and DAPI, over a 24 h period at normoxic or hypoxic conditions following manufacturer’s instruction. To avoid any effect of initial cell density equal numbers of cells that were precultured for a period of 14 day in normoxic or hypoxic conditions were used (8000 cells/cm2 seeded in Ibidi slides 80826 treated) and quantification using SlideBook 6 software (3i Intelligent Imaging Innovations, GmbH, Göttingen, Germany) occurred in six fields of duplicate wells using 3 different donors.

### Western Blot

15x10^4^ hMVECs (on 5 cm^2^) were washed with PBS and lysed in Laemmli Sample Buffer (Bio-Rad, Hercules, USA) with 5% β-mercaptoethanol. Protein was separated on an 8% SDS-polyacrylamide gel and electrophoretically transferred onto nitrocellulose membrane (Amersham, Uppsala, Sweden) in a buffer of 192 mM glycine, 25 mM Tris (pH 8.3) and 10% (v/v) methanol. The membranes were blocked with 5% (w/v) non-fat milk in 137 mM NaCl, 20 mM Tris (pH 7.6) and 0.1% Tween 20 (TBST) for 90 minutes, followed by overnight incubation at 4°C with the primary polyclonal antibodies (anti-HIF-1α 1:250 (Cayman chemical; 10006421), anti-HIF-2α 1:500 (Novus Biologicals; NB100-122)) in TBST + 5% non-fat milk. Subsequently, the blots were washed three times with TBST and incubated for 90 minutes at room temperature with horseradish-conjugated goat-anti-rabbit antibodies (1:5000) or horseradish-conjugated goat-anti-mouse antibodies (1:5000) (Dako, Darmstadt, Germany) in TBST + 5% nonfat milk as a conjugate. The bands were visualized with enhanced chemiluminescence (Sigma) on a LAS3000 machine (Fujifilm, Japan).

### RNA isolation and quantitative Real-time PCR

hMVECs were cultured for 14 days at normoxic or hypoxic conditions. Upon confluency, cells were starved for 18 hours in SSC medium and afterwards stimulated with VEGF (10 ng/mL) and TNFα (10 ng/ml) in SSC medium for 24 hours. Total RNA was isolated by using the RNeasy Mini kit according to manufacturer’s protocol without the DNAse treatment (Qiagen). Subsequently copy DNA (cDNA) was synthesized of 1 μg RNA using the Cloned AMV First Strand cDNA Synthesis Kit from Invitrogen with poly(T)primers. β-2-microglobulin was used as the endogenous reference gene. To measure gene expression, quantitative Real-time PCR (qRT-PCR) was performed in duplicate wells using SYBR Green in an ABI 7500 sequence detection system (Applied Biosystems, Foster City, USA). Briefly, 10 μl mix was prepared using 20 ng cDNA, 100 nM forward primer, 100 nM reverse primer and MESA Green QPCR Mastermix Plus for Sybr Assay (Eurogentec, Seraing, Belgium). Protocol: 2 min 50°C, 10 min 95°C and 40 cycles (0:15 min 95°C, 1:00 min 60°C) and dissociation curve. Relative expression levels of target genes (see also S1 Table) were calculated with the reference gene β-2-microglobulin with the comparative C_q_ method, as described by Wong *et al* [38].

### Statistical analysis

Statistical analysis was performed using repeated measures ANOVA or TWO-way ANOVA with Bonferroni post-hoc test. Numbers of replicates and significant P-values are indicated in the text or figures. P<0.05 was considered significant. Results are shown as mean ±SEM or ±SD.

## Results

### Generating hypoxic cell culture conditions

Human microvascular endothelial cells (hMVECs) isolated from the foreskin were precultured at ambient air/ 5% CO_2_ atmosphere (20% O_2_, further indicated as normoxia) or at 1% O_2_/ 5% CO_2_ (further indicated as hypoxia). The hypoxic conditions were maintained inside a custom-designed hypoxic workstation with CO_2_ and O_2_-controlled humidified incubators and a microscope inside the hypoxic workstation (S1 Fig). To prevent reoxygenation, culture media, enzyme solutions and buffers were transferred into the workstation via a lock and pre-incubated at 1% oxygen before use. As revealed by PreSens Oxo dish^®^ measurements, it takes several hours before oxygen levels of 500 μL (grey line; S2A Fig) in a 2 cm^2^ well drop from 20% to 1% oxygen. Moreover, the microscope inside the hypoxic station was used to monitor the cells, since a period of only 5 minutes at ambient air will increase the oxygen levels of a small volume of hypoxic medium (250 μL; black line; S2B Fig) to 10% O_2_, which causes rapidly reoxygenation of the cells.

### Human endothelial cells are viable and proliferate in prolonged hypoxia

**Fig 1:**
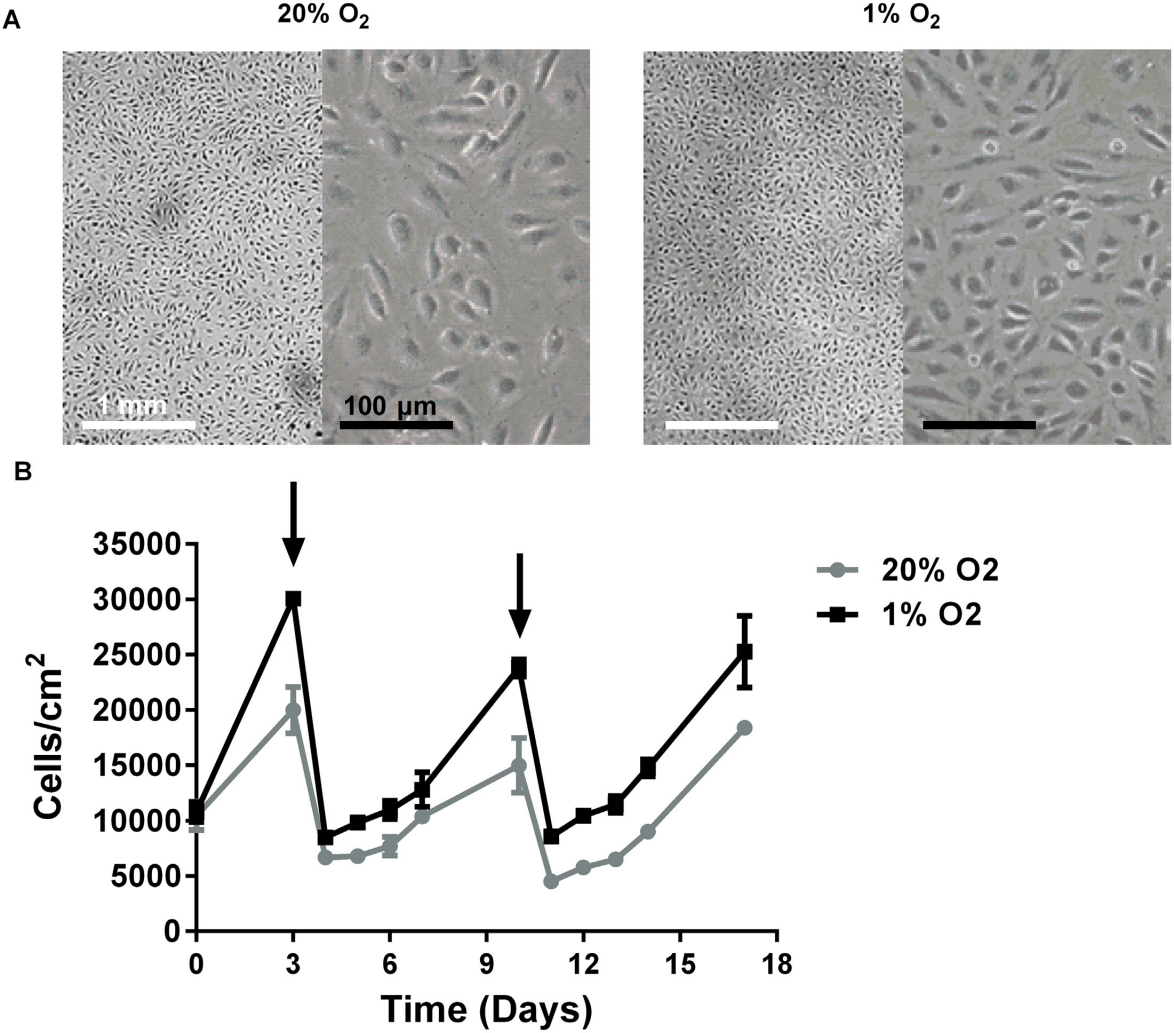
Effect of hypoxia on human microvascular endothelial cell proliferation. hMVECs were cultured in normoxia (20% O_2_, grey circles) or hypoxia (1% O_2_, black squares) for 17 days. **(A)** Photos of confluent monolayers of hMVECs on day 17 are shown. The white scale bar represents 1 mm and the black scale bar represents 0.1 mm. **(B)** Photos were taken every day and analyzed with ImageJ software, the arrows indicate days that cells were passaged 1:3. Representative experiment out of 3 independent hMVEC donors, showing number of cells/cm^2^ as average of triplicate wells with SD.

As a model for chronic hypoxia, endothelial cells were cultured for 2 passages (~14 days) in hypoxic conditions preceding the experiments. After 14 days of culturing in 1% oxygen hMVECs were still viable (>98% trypan blue exclusion) and formed a tight confluent monolayer (Fig 1A). To determine whether their proliferative capacity was altered during prolonged hypoxia, cell numbers were daily monitored for several passages in normoxic and hypoxic conditions during a period of 17 days. Depending on the cell isolation used, no difference or even a slight increase in cell number was found in hypoxic cultured cells compared with normoxic cultured cells (Fig 1B). Similarly, quantification of EdU incorporation as measurement of cell proliferation controlled by DAPI staining revealed no significant difference in proliferation capacity between normoxic and hypoxic precultured cells (45.6% ± 7.8 and 52.0% ± 9.1 in normoxic and hypoxic cultures, respectively, n = 3 donors, see S3 Fig).

### Prolonged hypoxia impairs endothelial sprout formation

**Fig 2:**
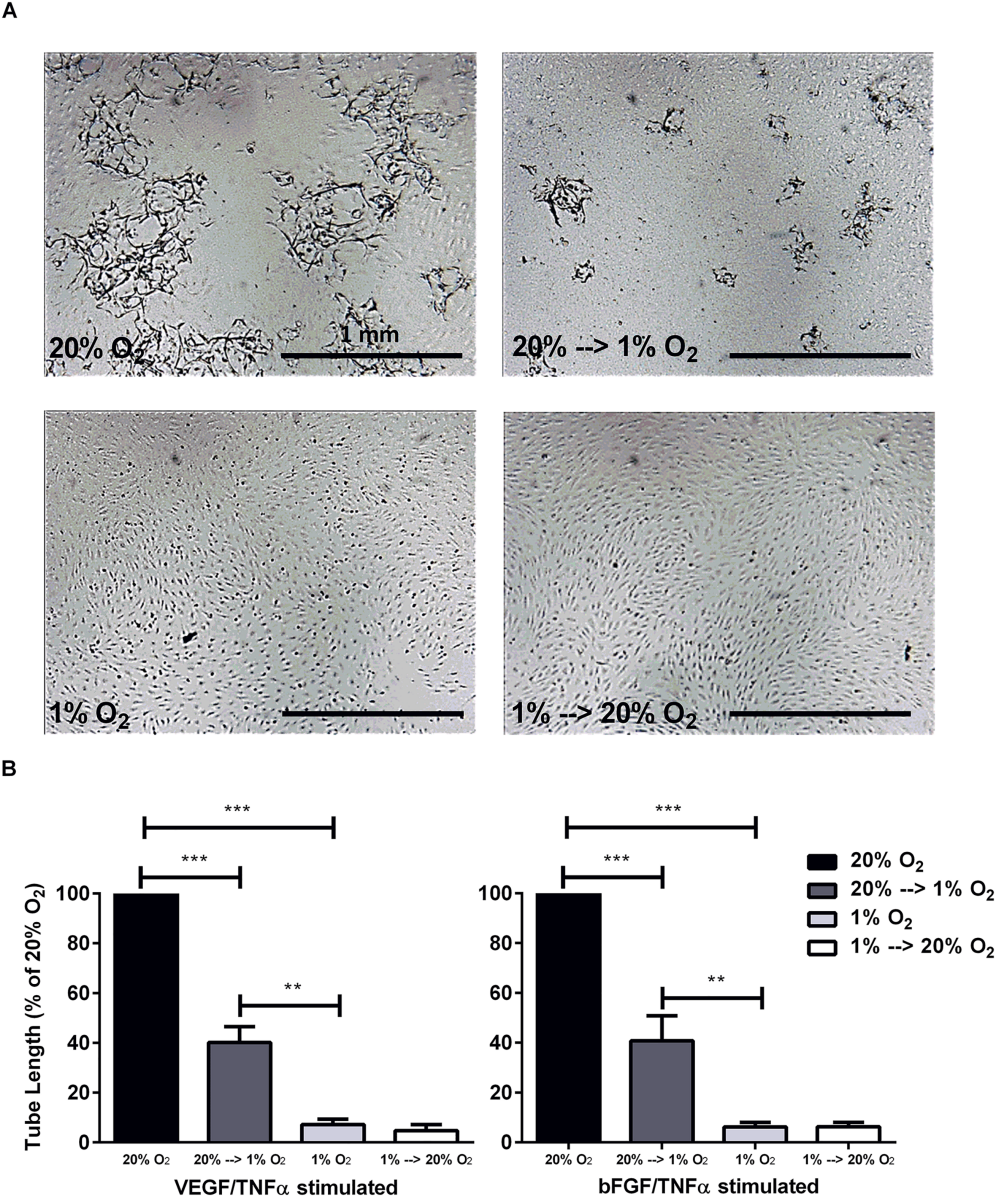
Prolonged hypoxia inhibits sprouting of endothelial cells into 3D fibrin matrices. hMVECs were precultured at 20% (black and dark grey bars) or 1% oxygen (light grey and white bars) for 14 days before seeded on top of 3D fibrin matrices. Subsequently, the hMVECs were stimulated with the combination of VEGF-A/TNFα (4 donors in 7 experiments) or bFGF/TNFα (5 donors in 10 experiments) either at 20% oxygen (black and white bars) or at 1% oxygen (dark grey and light grey bars) (each in quadruple). **(A)** Representative photos are shown of hMVECs 7 days after seeding and stimulation with VEGF-A/TNFα. Scale bars represent 1 mm. Photos are focused on the sprouts. **(B)** Tube length was quantified by using Optimas software and expressed as percentage of 20% O_2_ with SEM. For statistical analysis repeated measures ANOVA with Bonferroni post-hoc test was used (** p<0.01 *** p<0.001).

hMVECs, precultured for 14 days in normoxia or hypoxia, were seeded on top of 3D fibrin matrices and subsequently incubated at 20% O_2_ or 1% O_2_. The normoxic precultured cells formed sprouts in normoxia when stimulated with the combinations of VEGF-A/TNFα or bFGF/TNFα for 7 days (Fig 2A), whereas the normoxic precultured cells formed significantly less sprouts after 7 days in hypoxia upon VEGF-A/TNFα and bFGF/TNFα stimulation (60±6% and 59±10% inhibition respectively; Fig 2B). Sprout formation was even further inhibited when the cells were precultured in hypoxia and the experiment was performed in hypoxia upon VEGF-A/TNFα and bFGF/TNFα stimulation (93±2% and 94±2% inhibition respectively; Fig 2B). Moreover, the hypoxic precultured cells did not form sprouts in normoxia upon VEGF-A/TNFα and bFGF/TNFα stimulation (95±2% and 94±2% inhibition respectively).

### Regulation of HIFs in short-term and long-term hypoxia

**Fig 3:**
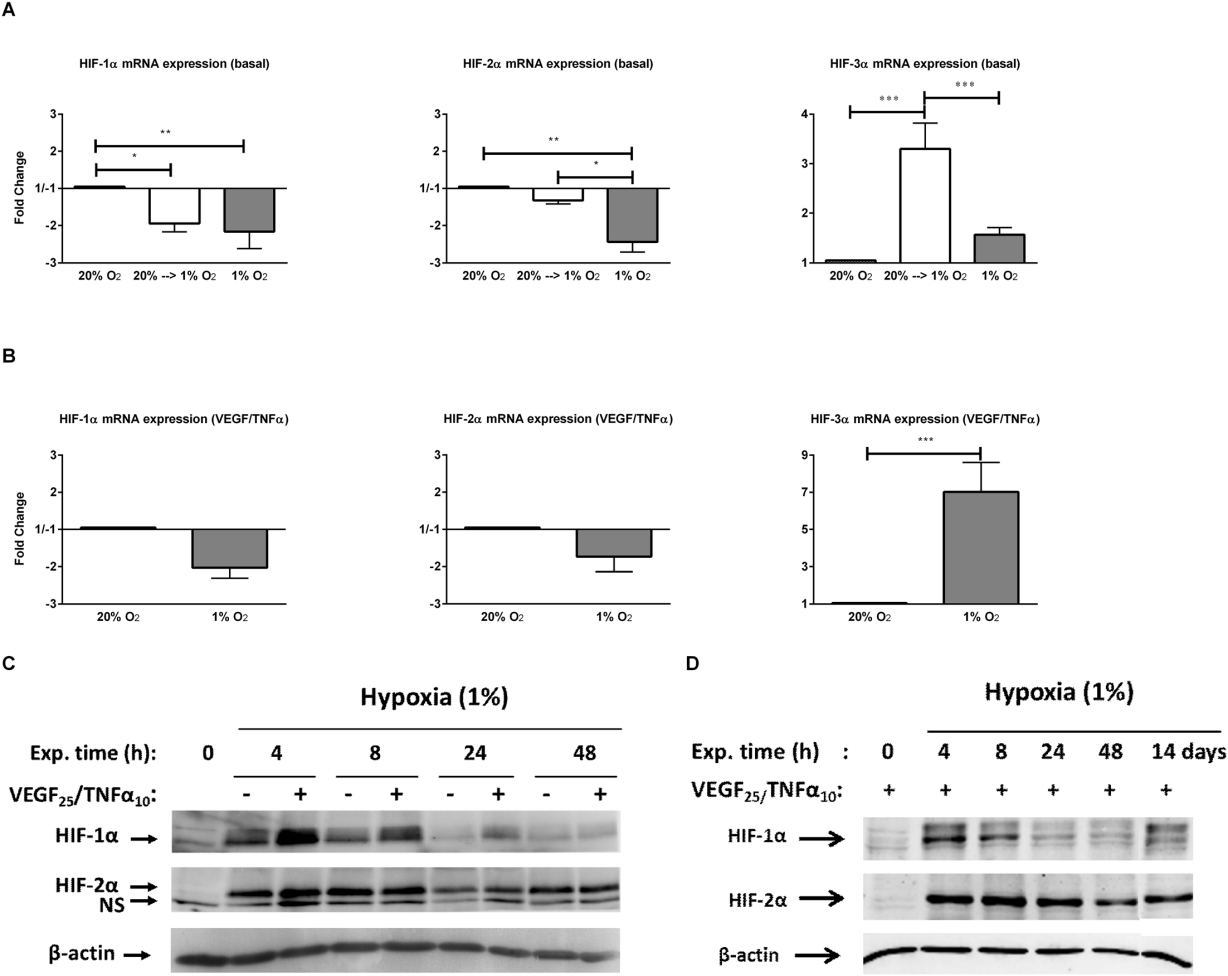
Effect of hypoxia on the regulation of HIF mRNA and protein. mRNA was isolated from hMVECs precultured for 14 days at 20% O_2_ that were incubated for 24 hours in 20% O_2_ (black bar) or in 1% O_2_ (white bar) or precultured for 14 days at 1% O_2_ and incubated for 24 hours in 1% O_2_ (grey bar). Cells were either **(A)** not stimulated (4 independent donors) or **(B)** stimulated with VEGF-A/TNFα for 24 hours (4 independent donors) and expressed as mean fold change with SEM. Data was normalized to 20% O_2_. **(C)** Western blot analysis of whole cells lysates collected from cells precultured at 20% O_2_ for 14 days and incubated in hypoxia with or without VEGF-A/TNFα for indicated hours (2 independent donors) or **(D)** western blot analysis of whole cell lysates collected from hMVECs precultured at 20% O_2_ for 14 days and incubated in hypoxia with VEGF-A/TNFα for indicated hours or hMVECs precultured in hypoxia for 14 days and stimulated with VEGF-A/TNFα for 24 hours (3 independent donors). For statistical analysis TWO-way ANOVA with Bonferroni post-hoc test was used (A and B) (* p<0.05 **p<0.01 *** p<0.001).

While in normoxic conditions HIF-1α and HIF-2α mRNA was abundantly expressed in hMVECs (C_q_ values 20.5±0.2 and 22.6±0.5 respectively), no HIF-1α or HIF-2α protein was detected by Western blotting (Fig 3C). Under non-stimulated conditions, HIF1A mRNA (further indicated with protein symbol HIF-1α) was significantly reduced after 24 hours of hypoxia and prolonged hypoxia (-1.9±0.2-fold and -2.2±0.5-fold respectively). Although 24 hours of hypoxia did not alter EPAS1 mRNA (further indicated with protein symbol HIF-2α) expression (-1.3±0.1-fold), the levels were significantly reduced in prolonged hypoxia (-2.4±0.3-fold, see Fig 3A). Upon stimulation with VEGF-A/TNFα, HIF-1α mRNA was reduced in prolonged hypoxia (-2.0±0.3-fold) and a similar trend was also observed for HIF-2α mRNA expression (-1.7±0.4-fold, Fig 3B). Despite the reduction on mRNA level, both HIF-1α and HIF-2α proteins were increased in non-stimulated cells after exposure to hypoxia, which was considerably higher (mainly HIF-1α protein) when VEGF-A/TNFα was present (Fig 3C). These increases were transient, in particular for HIF-1α, which resulted in a marked shift in the balance between HIF-2α and HIF-1α after 48 hours and 14 days of hypoxia (Fig 3D).

In contrast to HIF-1α and HIF-2α mRNA levels, hMVECs express low levels of HIF3A mRNA (further indicated with protein symbol HIF-3α) in normoxia (C_q_ values 29.3±0.6), which was significantly increased under non-stimulated conditions after 24 hours in hypoxia (3.3±0.5-fold). This increase was attenuated after a period of prolonged hypoxia (1.6±0.1-fold, Fig 3A). Upon VEGF-A/TNFα stimulation, however, HIF-3α mRNA was significantly increased in prolonged hypoxia (7.0±1.6-fold, Fig 3B).

### Silencing of HIF-2α with si-RNA restores sprouting in prolonged hypoxia

**Fig 4:**
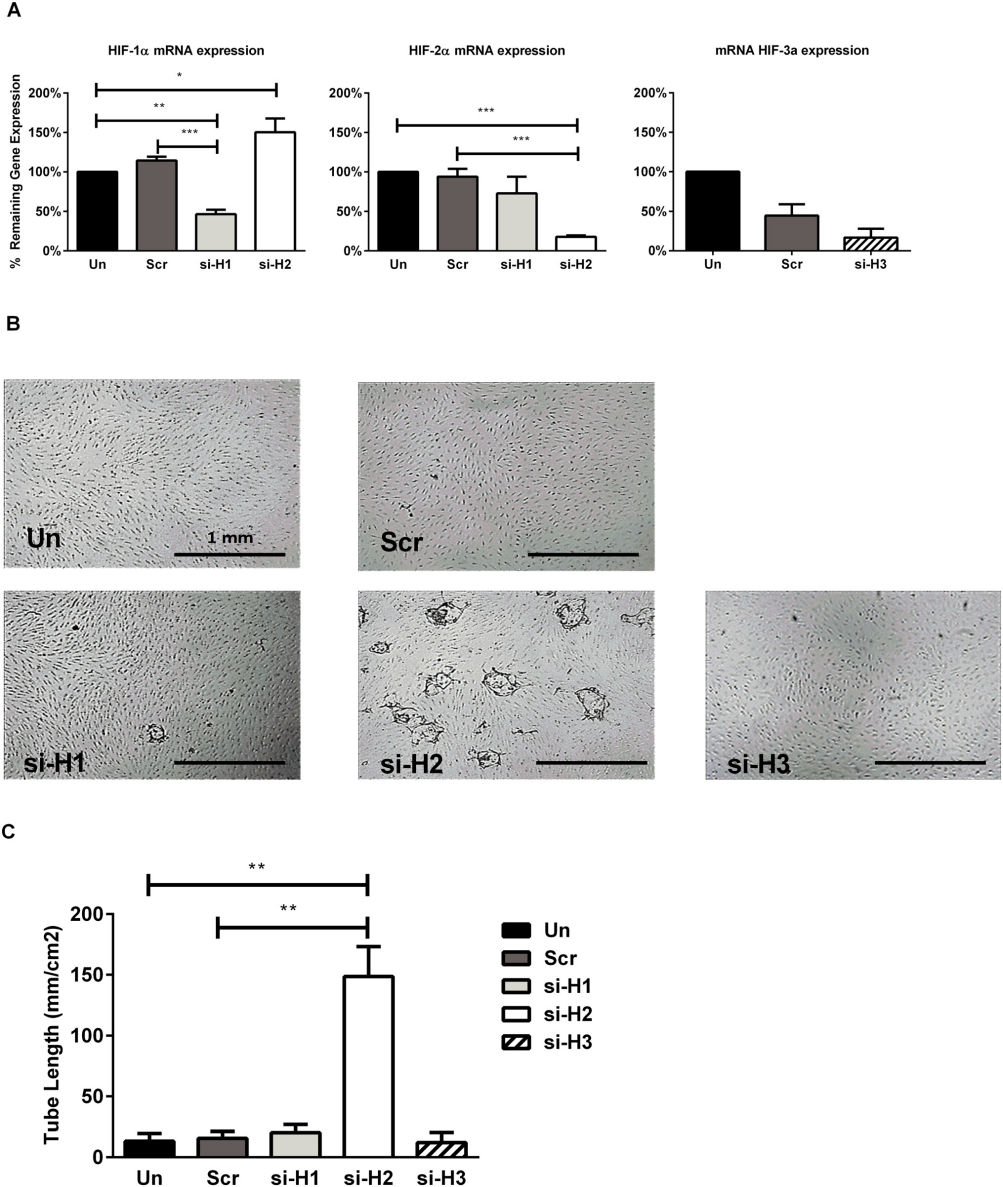
Knockdown of HIF-2α but not HIF-1α restores sprouting in prolonged hypoxia. hMVECs were precultured in hypoxia for 14 days and either untransfected (Un, black bar) or transfected with scrambled si-RNA (Scr, dark grey bar) or si-RNA against HIF-1α (si-H1, light grey bar), HIF-2α (si-H2, white bar) or HIF-3α (si-H3, hatched bar). Either mRNA was isolated to analyze the knock-down efficiency or the transfected hMVECs were seeded on top of 3D fibrin matrices. (A) The knock-down efficiency was expressed as mean with SEM (4 independent donors) and untransfected was set as 100%. (B) Representative photos are shown of hMVECs 7 days after seeding and stimulation with bFGF/TNFα. Scale bars represent 1 mm. Photos are focused on the sprouts. (C) The tube length was quantified by using Optimas software and expressed as mean tube length (in mm/cm^2^) with SEM (3 independent donors, each in quadruple). For statistical analysis repeated measures ANOVA with Bonferroni post-hoc test was used (* p<0.05 **p<0.01).

To investigate the roles of HIF-1α, HIF-2α and HIF-3α on (the inhibition of) endothelial sprouting, hMVECs were cultured in normoxia or prolonged hypoxia, transfected with si-RNAs before seeding on top of 3D fibrin matrices and stimulated with bFGF/TNFα or VEGF-A/TNFα. HIF-1α and HIF-2α were silenced by transfection with specific si-RNA in 20% oxygen (data not shown) and 1% oxygen (53±6% and 82±2% reduction respectively), whereas scrambled si-RNA did not silence HIF-1α or HIF-2α (14±6% induction and 6±10% reduction respectively; Fig 4A). Surprisingly, the HIF-2α-silenced cells showed a restored sprouting capacity in cells precultured in prolonged hypoxia (Fig 4B and 4C) upon bFGF/TNFα or VEGF-A/TNFα stimulation. This increase was significant compared with untransfected prolonged hypoxic precultured cells and prolonged hypoxic precultured cells transfected with the scrambled counterpart. Transfection with si-RNA of HIF-1α did not restore sprouting in prolonged hypoxia precultured (Fig 4B and 4C). Moreover, HIF-3α mRNA was almost completely silenced upon transfection with specific si-RNA (91%, Fig 4A), but the sprouting in prolonged precultured hypoxic cells was not restored after 7 days with VEGF-A/TNFα or bFGF/TNFα stimulation (Fig 4B and 4C).

### Effect of hypoxia and HIF-2α on VEGF-A and DLL4/NOTCH signaling

Two candidate pathways that were identified in animal studies as HIF-regulated mediators of endothelial sprouting are the VEGF/VEGF receptor pathway (regulated by HIF-1α) [18,19] and Dll4/Notch1 signaling pathway (regulated by HIF-2α) [20,21]. Under our experimental conditions neither DLL4 and NOTCH1, nor genes reflecting Dll4/Notch signaling, such as the members of the Hes and Hey family, were significantly affected by prolonged hypoxia in combination with VEGF-A/TNFα (Table 1). As previous Dll4/Notch studies [20,21] were performed in mouse lungs and cultured mouse lung microvascular endothelial cells, we subsequently cultured human lung MVECs of two donors separately and exposed them to 1% oxygen for 24 hours. Similar as in human foreskin MVECs, only a small increase in DLL4 and no increase in NOTCH1 mRNA were observed, with only minor effects on HEY1 and HEY2 mRNA expression. The VEGF-A mRNA expression was increased after a 24-hour exposure to hypoxia in human MVECs isolated from the foreskin or lungs, which was comparable to the VEGF-A levels found in mouse lungs. However, the mRNA expression of the FLT1 and KDR (further indicated with protein symbol VEGFR-1/2) was not consistently changed in response to hypoxia (Table 1). This indicates that in human endothelial cells, hypoxia marginally changed the expression of the Dll4/Notch1 or VEGF/VEGF receptor system and that under our experimental conditions the activation of these pathways seem unlikely to be responsible for the inhibitory effect of prolonged hypoxia on endothelial cell sprouting.

**Table 1:** Effect of hypoxia on relative mRNA expression (hypoxia/normoxia) of DLL4-NOTCH signaling genes in different human microvascular endothelial cells.

Relative mRNA expression		
Gene	hMVEC prolonged VT	Lung hMVEC 24h basal
**VEGFA**	3.49±0.22 ***	14.09±1.20
**FLT1**	0.89±0.12	2.26±0.99
**KDR**	0.85±0.10	0.43±0.97
**NOTCH1**	0.93±0.04	0.71±0.06
**DLL4**	0.93±0.11	1.37±0.47
**HES1**	1.44±0.24	1.67±0.30
**HES2**	1.03±0.08	ND
**HEY1**	1.19±0.31	1.04±0.33
**HEY2**	0.64±0.11 *	1.53±1.43

Relative mRNA levels were expressed as mean fold change with SEM (column 2) or range (column 3) (relative to normoxic controls). **Column 2** Human foreskin microvascular endothelial cells (hMVECs) were precultured for 14 days in 1% oxygen (prolonged) and stimulated for 24 hours with VEGF-a/TNFα (4 independent donors). **Column 3** Human lung microvascular endothelial cells (lung hMVECs) were precultured at 20% oxygen and incubated for 24 hours in hypoxia under basal conditions (2 independent donors). ND: not determined. (* p<0.05 *** p<0.001).

### The uPA/uPAR/plasmin pathway is affected in prolonged hypoxia but independent of HIF activation

**Fig 5:**
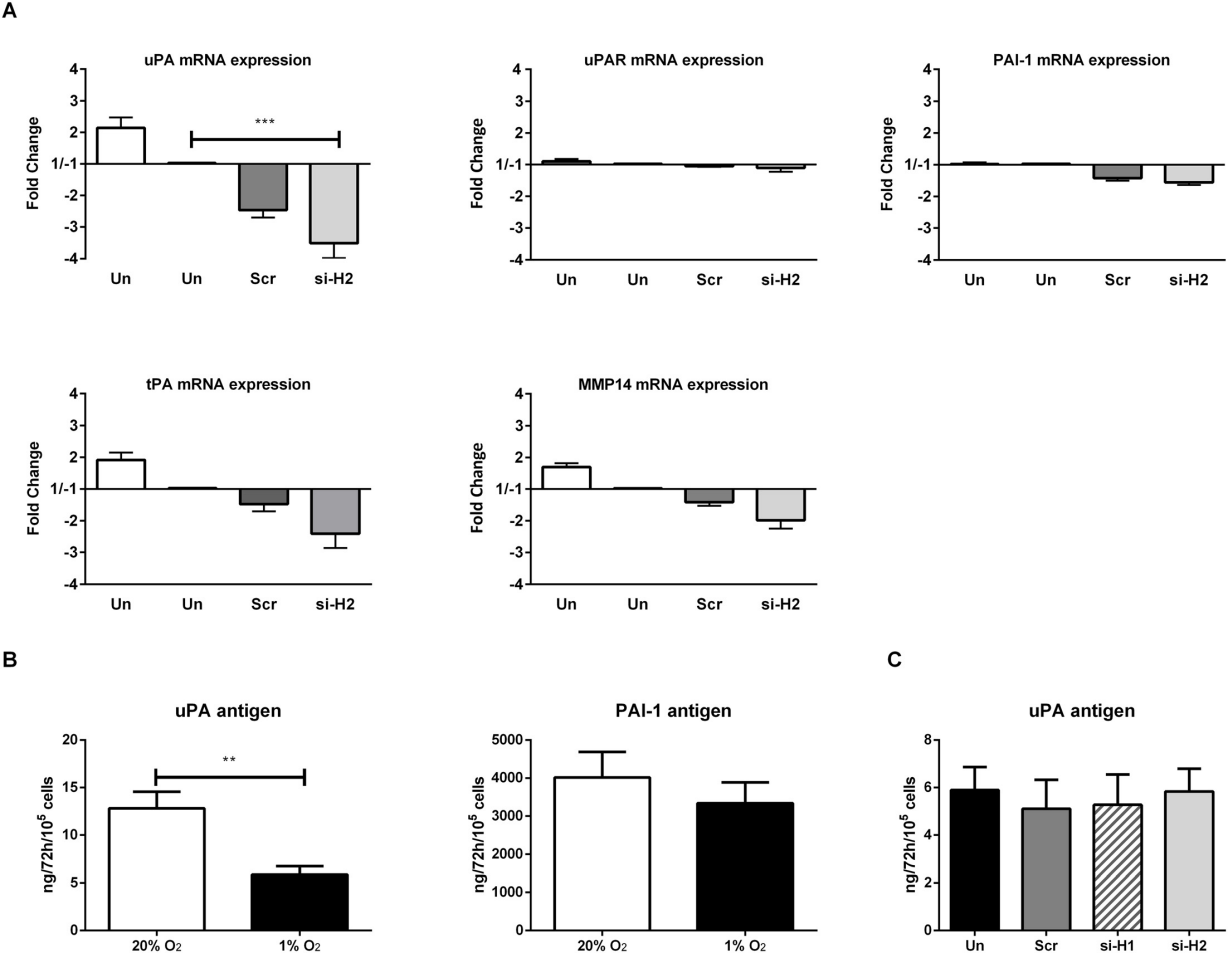
Effect of prolonged hypoxia on the expression of genes involved in pericellular proteolysis. **(A)** hMVECs were cultured in normoxia (Un (20%), white bar) or prolonged hypoxia (Un (1%), black bar) for 14 days and transfected with si-HIF-2α (si-H2 (1%), light grey bar) or scrambled (scr (1%), dark grey bar) and stimulated for 24 hours with VEGF/TNFα. mRNA was isolated for analysis by qRT-PCR and the relative mRNA levels were expressed as mean fold change with SEM (4 independent donors). Data was normalized to 1% O_2_. **(B)** uPA and PAI-1 antigens were measured in hMVECs cultured in 20% O_2_ (white bar) or 1% O_2_ (black bar). Levels were expressed as ng/72h/10^5^ cells with SEM (4 independent donors). **(C)** uPA antigen was measured in hMVECs cultured in 1% O_2_ and either untransfected (Un, black bar), or transfected with scrambled si-RNA (scr, dark grey bar), si-HIF-1α (si-H1, hatched bar), or si-HIF-2α (si-H2, light grey bar). Levels were expressed as ng/72h/10^5^ cells with SEM (3 independent donors). For statistical analysis TWO-way ANOVA with Bonferroni post-hoc test (A), paired student t test (B), or repeated measures ANOVA with Bonferroni post-hoc test was used (C) (** p<0.005, *** p<0.001).

The uPA/uPAR/plasmin system is a pivotal enzyme system involved in endothelial sprouting into fibrin [24,39]. In normoxia, the sprouting of endothelial cells was blocked by silencing uPA or uPAR with si-RNA (S4 Fig), while si-SERPINE1 (plasminogen activator inhibitor-1 (PAI-1)) either enhanced tube formation 2-fold or caused cell detachment, depending on the hMVEC donor used (data not shown). This underlines the importance of the uPA/uPAR system in our model. Prolonged hypoxia reduced the mRNA expression of uPA, although not significantly in VEGF-A/TNFα stimulated cells (-2.3±0.7, Fig 5A). Moreover, the mRNA expression of uPAR, PAI-1 or MMP-14 did not change (1.2±0.2-, 1.0±0.1- and 1.7±0.2-fold respectively) in this hypoxic condition. Similarly, the accumulation of uPA antigen was significantly decreased by prolonged hypoxia (from 12.8±1.8 to 5.85±0.9 ng/72h/10^5^ cells, Fig 5B), while PAI-1 antigen was only altered to a minor extent (4016±674 vs 3341±546 ng/72h/10^5^ cells, Fig 5B). Blockage of uPA binding to uPAR by MoAb H-2 increased the accumulation of uPA to the same extent in hypoxic and normoxic hMVECs (ΔuPA antigen: 1.85±0.2 vs 1.9±0.0.25 ng/72h/10^5^ cells, respectively; 2 independent donors, data not shown), suggesting that the overall internalization of the uPA:PAI-1 complex via uPAR was not significantly affected.

The reduction in the uPA expression may explain the inhibition of sprouting in prolonged hypoxia. However, silencing of HIF-2α, which restored sprouting (Fig 4), did not restore, but even further decreased, the expression of uPA mRNA upon VEGF-A/TNFα stimulation (Fig 5A). Neither the uPAR nor the PAI-1 mRNA expression was significantly altered by silencing HIF-2α (-1.2±0.2-fold and -1.6±02-fold respectively). Furthermore, neither si-HIF-2α nor si-HIF-1α changed the accumulation of uPA antigen (99±4% and 87±8% of control, respectively; Fig 5C) in hypoxic hMVECs, suggesting that hypoxia exerts two effects: one which involves downregulation of uPA, and one that involves expression of HIF-2α but occurs without further affecting uPA-mediated pericellular proteolysis.

**Fig 6:**
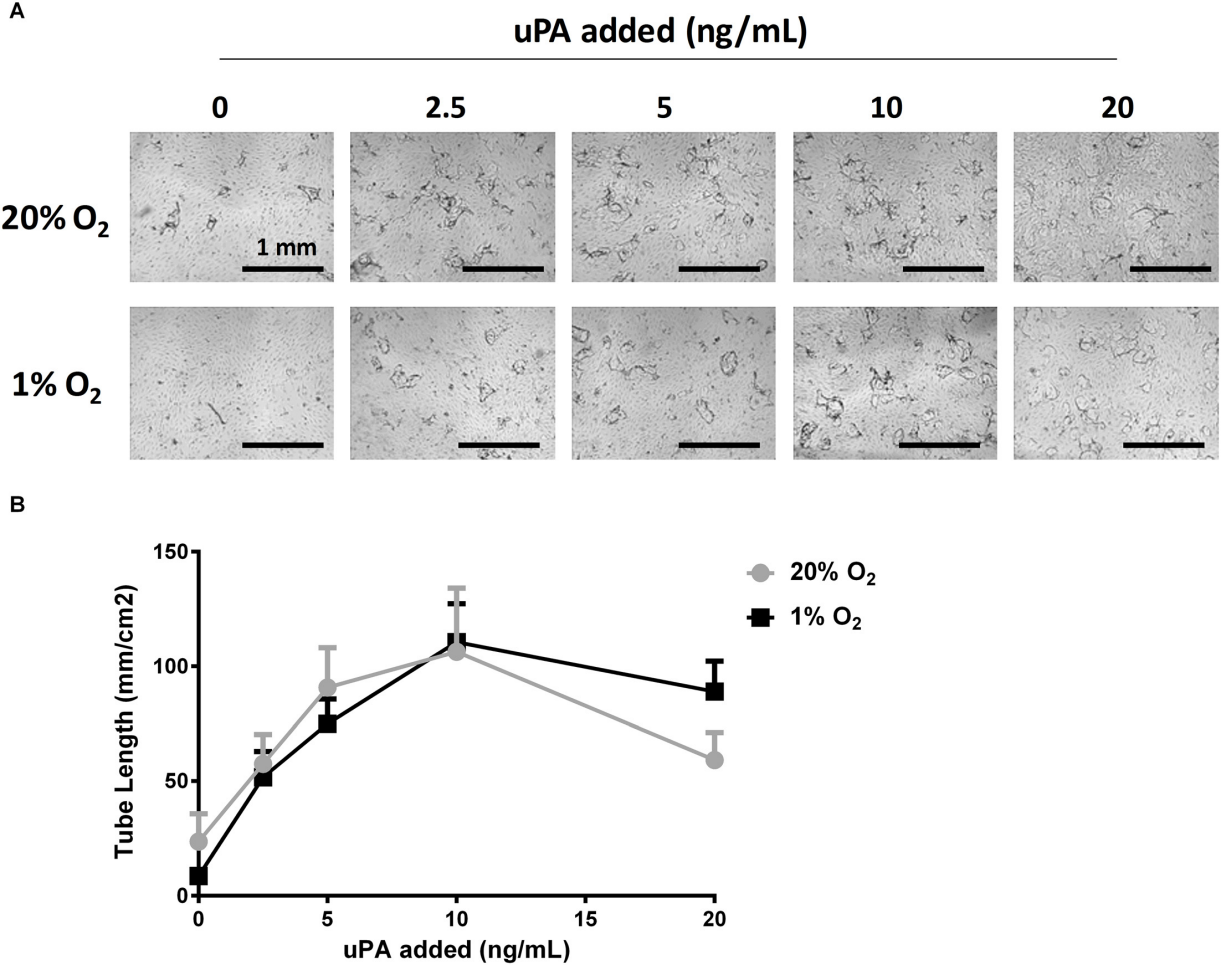
Addition of recombinant uPA increases endothelial sprouting. hMVECs were seeded on top of 3D fibrin matrices and stimulated with the combination of VEGF-A/TNFα and the indicated concentration of human recombinant uPA either in normoxia (grey circles) or hypoxia (black squares) (each in quadruple). **(A)** Representative photos are shown of hMVECs 7 days after seeding and stimulation with VEGF-A/TNFα. Scale bars represent 1 mm. Photos are focused on the sprouts. **(B)** Tube length of a representative experiment was quantified by using Optimas software and expressed as mm/cm^2^ with SEM. After addition of 20 ng/ml u-PA lysis of fibrin was observed, which interfered with the stability of the tubular structures.

To evaluate whether addition of exogenous uPA could overcome the sprouting inhibition of prolonged hypoxia, we added different concentrations of human recombinant-uPA to the medium during the sprouting assay. Addition of 2.5–10 ng/ml hr-uPA markedly increased tube formation in hypoxia, as well as in normoxia (Fig 6A and 6B). At higher u-PA concentration (20 ng/ml) lysis of fibrin was observed, which interfered with the stability of the tubular structures.

## Discussion

The present study showed that, unlike short-term hypoxia, prolonged hypoxia (14 days preculturing and subsequent incubation at 1% oxygen) completely blocked the ability of hMVECs at 20% oxygen to form sprouts into a 3D fibrin matrix upon VEGF-A/TNFα stimulation, although the cells were viable. The inhibition of sprouting during long-term hypoxia was partially restored when endothelial cells were silenced for HIF-2α, but not for HIF-1α or HIF-3α. The reduced capacity of the endothelial cell sprouting could not be attributed to changes in the Dll4/Notch1 signaling pathway. In addition, hypoxia caused a decrease in uPA production; however, this effect was not influenced by HIF-1α or HIF-2α. As inhibition of uPA causes reduction of sprout formation, and addition of exogenous uPA enhanced it, reduced uPA production probably contributes—in addition to the HIF-2α-dependent mechanism—to the hypoxia induced inhibition of endothelial tube formation.

### Prolonged hypoxia impairs endothelial sprout formation

hMVECs that were precultured for 14 days at 1% oxygen were unable to form sprouts into a 3D fibrin matrix, in spite of the stimulation with VEGF-A/TNFα. Although we showed that after 14 days hMVECs proliferated in a similar rate in normoxia and hypoxia, the TNFα that was added during the sprouting assay inhibited proliferation of the endothelial cells [24]. Therefore, during the sprouting assay, tube formation is largely driven by migration and invasion. hMVECs, precultured in normoxia and exposed to 1% oxygen for 7 days, formed sprouts but significantly less compared with endothelial cells that were only exposed to 20% O_2_. This is contradictory to the general thought that hypoxia is a stimulator of angiogenesis [40,41]. As most *in vitro* studies only investigate the effect of short-term hypoxia on sprouting, this could explain the stimulation generally found [2,4,32,33]. To the best of our knowledge, limited data are presently available on endothelial sprouting during prolonged hypoxia; it was only shown that an exposure to hypoxia for more than 7 days significantly reduced the capillary formation of endothelial cells [7,8]. However, these cells were precultured at 20% oxygen. However, the hMVECs are stimulated with TNFα for 24 hours and little proliferation occurs in endothelial cells stimulated with TNFα [24].

### Regulation of HIFs in hypoxia

The reduced angiogenic capacity of microvascular endothelial cells after a prolonged hypoxic culturing could be due to a shift in the balance between HIF-1α and HIF-2α. Even though HIF-1α and HIF-2α proteins were quickly stabilized in response to hypoxia in hMVECs, HIF-1α has the highest expression between 4 and 8 hours in hypoxia, whereas HIF-2α protein levels remained high after 48 hours in hypoxia. This difference remained upon VEGF-A and TNFα stimulation, which markedly enhanced HIF-1α in particularly at 4 and 8 hours in hypoxia. This increase in HIF proteins in hypoxia was mainly caused by protein stabilization, as mRNA levels of HIF-1α and HIF-2α decreased in hypoxia (Fig 3). A similar mRNA and protein expression pattern of HIF-1α and HIF-2α was found in human umbilical cord veinendothelial cells (HUVECs) or other cell types [23,42,43]. Thus although some HIF-1α protein was present in long-term hypoxia, HIF-2α was the most dominantly expressed HIF in human microvascular endothelial cells exposed to prolonged hypoxia, which is in line with published data [9,44,45]. Ginouvès *et al* [23] suggested that the decreased HIF-1α protein expression after 24 hours in hypoxia was due to an increased expression and activity of PHDs in prolonged hypoxia. As we also found that PHD2 and PHD3 mRNA expression was increased in short-term and long-term hypoxia (our unpublished data), a feedback loop through the increased expression of PHD2 or PHD3 could regulate both HIF-1α and HIF-2α expression. Moreover, the transcriptional activity of HIF-1α followed a similar trend as the HIF-1α protein expression pattern [23,46]. In line with this, we found that the HIF-1α protein expression correlated with the HIF-3α mRNA expression, which is thought to be a HIF-1α-target gene [42,47,48]. An upregulation of HIF-3α mRNA in response to hypoxia was also shown in HUVECs [42] and several tissues in rodents [47,49]. However, these studies only investigated the effect of short-term hypoxia (up to 48 hours).

### Silencing of HIF-2α with si-RNAs partly restores sprouting in prolonged hypoxia

The inhibited endothelial sprouting under prolonged hypoxic conditions was partially restored upon silencing of HIF-2α, but not by silencing of HIF-1α indicating that HIF-1α and HIF-2α have different functions in human endothelial sprouting. HIF-1α stimulates angiogenesis-related processes such as endothelial sprouting and proliferation [20,50–52], whereas HIF-2α stimulates vessel remodeling into mature and functional vessels [20,21,53,54] or strengthening of the endothelial barrier [22]. To the best of our knowledge, our findings indicate for the first time that HIF-2α also restrains endothelial sprouting in a human setting, as found *in vivo* in animal studies [20,55,56].

The third HIF-α protein, HIF-3α, has been reported to act—as alternatively spliced variants—as a competitive inhibitor of HIF-1α and HIF-2α [49,57,58] and could both stimulate [32] and inhibit angiogenesis [49] in mice. Whether HIF-3α regulates angiogenesis in humans is not known. Our study indicated that even though HIF-3α mRNA was significantly increased in response to hypoxia, we, like others [42], were unable to detect HIF-3α on protein level in hMVECs or HUVECs. Moreover, silencing of HIF-3α did not restore sprouting. Therefore, as HIF-3α protein levels were only detected in the lungs and brains of rodents [32], this points toward a species- or tissue-specific function of HIF-3α.

### NOTCH signaling is not altered in prolonged hypoxia

Two candidate pathways for the inhibition of sprouting in prolonged hypoxia are the HIF-1α-regulated VEGF/VEGFR signaling pathway [18,19] and the HIF-2α-regulated Dll4/Notch1 signaling pathway [20,21,56,59]. Our study showed that hypoxia increased the mRNA expression of VEGF-A in human MVECs isolated from the lung or foreskin to a similar extent as found in mice lungs [20,21]. However, this did not result in a similar increase in VEGFR-1 and VEGFR-2 mRNA expression in human MVECs as was found in mouse cells. Moreover, no or only slight changes in mRNA levels of DLL4 and Notch-signaling genes (HES1/2 and HEY1/2) were found in our human lung and foreskin MVECs in response to hypoxia, whereas these genes were increased (e.g. DLL4 was increase 15-fold) in mouse endothelial cells and mouse lung [20,21]. It is not yet known whether this difference is related to species specificity. As it is unlikely that the involvement of these major pathways is different in man and mouse, it suggests that the response to hypoxia reveals differences in the extent to which the Dll4/Notch pathway is activated.

### Prolonged hypoxia reduces uPA production

Local pericellular proteolysis is important to achieve endothelial cell matrix invasion and sprouting [60]. We and other investigators [24,39,61] have shown that pericellular proteolysis by the uPA/uPAR/plasmin system plays a pivotal role in endothelial sprouting into fibrin. Sprouting of endothelial cells was inhibited by anti-uPA or anti-uPAR antibodies [26,33] and similar results were found in the present study upon silencing of uPA or uPAR with si-RNA. Furthermore, the addition of uPA could compensate for the decrease in uPA antigen and markedly enhanced endothelial tube formation. However, as recombinant uPA was used, a direct comparison of the quantification of this effect with that by endogenous uPA should be made with caution.

Other investigators have shown that—in the absence of plasminogen—MMP14 acts as a fibrinolysin and is able to stimulate pericellular proteolysis [27,28]. However, in our experimental conditions the uPA/uPAR/plasmin system is the far dominant proteolytic pathway enabling invasion into the fibrin matrix [26]. As a consequence, the effect of uPA on sprouting in prolonged hypoxia need further *in vivo* underpinning.

### Manipulating downstream targets of HIF-2α to regulate endothelial sprouting

The present study showed in human cells that, in addition to uPA reduction, HIF-2α restrains endothelial sprouting; the inhibited endothelial sprouting after prolonged hypoxic culturing, was partially restored upon silencing of HIF-2α in combination of VEGF-A and TNFα. Moreover, HIF-2α plays an important role in stimulating vessel remodeling and strengthening of the endothelial barrier. This indicates that both the initiation and the maturation of the endothelial sprout into a functional vessel are regulated by HIF-2α. As these actions are probably regulated by different HIF-2α-downstream targets, it is important to investigate which targets influence which pathways. Therefore, manipulating specific downstream targets of HIF-2α provides a new to be further evaluated perspective for restoring reduced neovascularization in several pathological conditions, such as diabetic, ulcers or other chronic wounds, or for improvement of vascularization of implanted tissue-engineered scaffolds.

## Supporting Information

S1 Fig Hypoxic workstation. The workstation has an airtight workbench with continuous control of pO_2_ and temperature. The incubators have continuous control of pO_2_, pCO_2_, temperature and humidity. A microscope is placed inside the hypoxic station with a connection to the computer, which allows hypoxic culturing for weeks without re-oxygenation.
(TIF)

S2 Fig Time course of O_2_ tension at the bottom of the wells after changing the ambient oxygen environment from 20% to 1% oxygen. **(A)** The drop in oxygen levels over time of medium pre-incubated in 20% oxygen and transferred to 1% oxygen atmosphere (in 94% N_2_ and 5% CO_2_) when different amounts of culture medium were used in a 2 cm^2^ well. **(B)** Either 250 μL (black line) or 500 μL (grey line) was incubated in a 2 cm^2^ plate and medium was changed at t = 0h from 20% to 1% O_2_ (left) or 1% to 20% O_2_ (right). While it takes 2 hours for medium containing 20% O_2_ to reach a hypoxic level after transfer to 1% O_2_, a short incubation at 20% O_2_ increases the oxygen level in medium rapidly from 1% to above 5% O_2_. Note that the measurements started 5 minutes after incubation.
(TIF)

S3 Fig Effect of hypoxia on human microvascular endothelial cell proliferation (EdU incorporation). hMVECs were precultured in normoxia (20% O_2,_ white bar) or hypoxia (1% O_2,_ black bar) for 14 days. (A) Photos of EdU-positive (green fluorescent), proliferating hMVECs and cell nuclei are visualized with DAPI (blue) at 1% or 20% oxygen levels. (B) The number of EdU-positive hMVECs was quantitated using SlideBook 6 software and expressed as mean % EdU positive cells ± SD of 3 independent hMVEC donors. For statistical analysis paired student t test was used.
(TIF)

S4 Fig Knockdown of uPA or uPAR impairs sprouting in normoxia. hMVECs were precultured at 20% oxygen and transfected with si-RNA before seeded on top of 3D fibrin matrices. **(A)** Representative photos are shown of hMVECs 7 days after seeding and stimulated with VEGF-A/TNFα (2 independent donors, each in quadruple). Scale bars represent 1 mm. Photos are focused on the sprouts. **(B)** hMVECs were either untransfected (black bar), transfected with scrambled (dark grey bar), si-uPA (light grey bar), si-uPAR (white bar). Tube length was quantified by using Optimas software and expressed as relative tube length (as % of Un) with SD.
(TIF)

S1 Table Primer sequences for qRT-PCR. (PDF)
