# Correction: HIF-2α Expression Regulates Sprout Formation into 3D Fibrin Matrices in Prolonged Hypoxia in Human Microvascular Endothelial Cells

**DOI:** 10.1371/journal.pone.0163840

**Published:** 2016-09-22

**Authors:** Tessa D. Nauta, Monique C. A. Duyndam, Ester M. Weijers, Victor W. M. van Hinsbergh, Pieter Koolwijk

The fourth author's name is spelled incorrectly. The correct name is: Victor W. M. van Hinsbergh. The correct citation is: Nauta TD, Duyndam MCA, Weijers EM, van Hinsbergh VWM, Koolwijk P (2016) HIF-2α Expression Regulates Sprout Formation into 3D Fibrin Matrices in Prolonged Hypoxia in Human Microvascular Endothelial Cells. PLoS ONE 11(8): e0160700. doi:10.1371/journal.pone.0160700

## References

[1] Nauta TD, Duyndam MCA, Weijers EM, van Hinsbergh VMW, Koolwijk P (2016) HIF-2α Expression Regulates Sprout Formation into 3D Fibrin Matrices in Prolonged Hypoxia in Human Microvascular Endothelial Cells. PLoS ONE 11(8): e0160700 doi: 10.1371/journal.pone.0160700 27490118 10.1371/journal.pone.0160700 PMC4973926

