# Supplementary material for: HIF-2α Expression Regulates Sprout Formation into 3D Fibrin Matrices in Prolonged Hypoxia in Human Microvascular Endothelial Cells
Source: PLoS One. 2016 Aug 4;11(8):e0160700. doi: 10.1371/journal.pone.0160700 (PMC4973926; doi:10.1371/journal.pone.0160700)
Supplement: S1 Table — (PDF) [file pone.0160700.s005.pdf]

S1 Table. Primer sequences for qRT-PCR

Protein name	Gene Symbol	Forward Primer	Reverse Primer
**β-2-microglobulin** [1]	B2M	TTTCATCCATCCGACATTG	CGGCAGGCATACTCATCTTT
**HIF-1α** [2]	HIF1A	CCAGTTAGGTTCCTTCGATCAGT	TTTGAGGACTTGCGCTTTCA
**HIF-2α** [2]	EPAS1	AGCAGATGGACAACTTGTACCTGA	TGTCGCCATCTTGGGTCAC
**HIF-3α** [3]	HIF3A	AGAGAACGGAGTGGTGCTGT	ATCAGCCGGAAGAGGACTTT
**DLL4** [1]	DLL4	CGTCTGCCTTAAGCACTTCC	GAAATTGAAGGGCAGTTGGA
**NOTCH1** [1]	NOTCH1	CAGGGTGTGCACTGTGAGAT	GACAGGCACTCGTTGACATC
**VEGF-A** [4]	VEGFA	CTTGCCTTGCTGCTCTACCTCC	CATCCATGAACTTCACCACTTCGT
**VEGFR1** [4]	FLT1	ATCATTCCGAAGCAAGGTGTGAC	TCCTTCTATTATTGCCATGCGCT
**VEGFR2** [4]	KDR	TGGGAACCGGAACCTCACTATC	GTCTTTTCCTGGGCACCTTCTATT
**HES1** [1]	HES1	TCCGGAGCTGGTGCTGAT	TCCAGGACCAAGGAGAGAGGTA
**HEY1** [5]	HEY1	AACTGTTGGTGGCCCTGAATC	AATTCTTTGTGTTGCTGGGG
**HEY2** [5]	HEY2	TTCAAGGCAGCTCGGTAACT	GGGCATTTTACTTCCCCAAT
**uPA** [6]	PLAU	ACTACTACGGCTCTGAAGTCACCA	GAAGTGTGAGACTCTCGTGTAGAC
**uPAR** [7]	PLAUR	CATGCAGTGTAAGACCAACGGGGA	AATAGGTGACAGCCCGGCCAGAGT
**tPA** [6]	PLAT	CCAGATCGAGACTCAAAGCC	GACCCATTCCCAAAGTAGCA
**PAI-1** [6]	SERPINE1	GCACAACCCCACAGGAAC	TGCTTCAAACTTCTCTCCCAG
**MMP14** [8]	MMP14	GCAGAAGTTTTACGGCTTGCAA	CCTTCGAACATTGGCCTTGAT

References

1. Weijers EM, van Wijhe MH, Joosten L, Horrevoets AJG, de Maat MPM, van Hinsbergh VWM, et al. Molecular weight fibrinogen variants alter gene expression and functional characteristics of human endothelial cells. J Thromb Haemost. 2010;8: 2800–9. doi:10.1111/j.1538-7836.2010.04096.x

2. Calvani M, Rapisarda A, Uranchimeg B, Shoemaker RH, Melillo G. Hypoxic induction of an HIF-1alpha-dependent bFGF autocrine loop drives angiogenesis in human endothelial cells. Blood. 2006;107: 2705–12. doi:10.1182/blood-2005-09-3541

3. Heidbreder M, Fröhlich F, Jöhren O, Dendorfer A, Qadri F, Dominiak P. Hypoxia rapidly activates HIF-3alpha mRNA expression. FASEB J. 2003;17: 1541–3. doi:10.1096/fj.02-0963fje

4. Smadja DM, Bièche I, Uzan G, Bompais H, Muller L, Boisson-Vidal C, et al. PAR-1 activation on human late endothelial progenitor cells enhances angiogenesis in vitro with upregulation of the SDF-1/CXCR4 system. Arterioscler Thromb Vasc Biol. 2005;25: 2321–7. doi:10.1161/01.ATV.0000184762.63888.bd

5. Williams CK, Li J-L, Murga M, Harris AL, Tosato G. Up-regulation of the Notch ligand Delta-like 4 inhibits VEGF-induced endothelial cell function. Blood. 2006;107: 931–9.

doi:10.1182/blood-2005-03-1000

6. Houard X, Rouzet F, Touat Z, Philippe M, Dominguez M, Fontaine V, et al. Topology of the fibrinolytic system within the mural thrombus of human abdominal aortic aneurysms. J Pathol. 2007;212: 20–8. doi:10.1002/path.2148

7. Li Y, Sarkar FH. Down-regulation of invasion and angiogenesis-related genes identified by cDNA microarray analysis of PC3 prostate cancer cells treated with genistein. Cancer Lett. 2002;186: 157–64.

8. Muñoz-Nájar UM, Neurath KM, Vumbaca F, Claffey KP. Hypoxia stimulates breast carcinoma cell invasion through MT1-MMP and MMP-2 activation. Oncogene. 2006;25: 2379–92. doi:10.1038/sj.onc.1209273
